# Editorial: Community series in reassessing the immune system contribution in multiple sclerosis: therapeutic target, biomarkers of disease and immune pathogenesis, volume II

**DOI:** 10.3389/fimmu.2024.1505829

**Published:** 2024-11-01

**Authors:** Maria Teresa Cencioni, Luisa Maria Villar, Roberta Magliozzi

**Affiliations:** ^1^ Department of Brain Sciences, Imperial College London, London, United Kingdom; ^2^ Department of Immunology, Hospital Universitario Ramon y Cajal, Madrid, Spain; ^3^ Department of Neurosciences, Biomedicine and Movement Sciences, Universita’di Verona, Verona, Italy

**Keywords:** multiple sclerosis, disease modified therapies, immune system, biomarkers, Epstein-Barr virus (EBV)

Recent and advanced methodologies have enhanced the understanding of the immune system features in multiple sclerosis (MS), a chronic inflammatory disease of the central nervous system, facilitating the development of personalised therapies. The current collection of articles offers new insights into MS pathology and immunology, identifying therapeutic targets and biomarkers. The articles explore various topics, including immunological biomarkers for predicting treatment success, risks of developing secondary autoimmune conditions, PML, and resistance to disease-modifying therapies. The studies also contribute to elucidating some of the pathological roles of cytotoxic B and T cells, dysfunctional humoral regulatory immunity, and inflammatory cascades triggered by EBV reactivation in the CNS. Additionally, the Research Topic highlights emerging biomarkers of disease activity, offering a comprehensive view of the latest advancements in MS research. Out of the submitted works, nineteen articles were accepted and included in this Research Topic. Below, we outline the key findings from each study.

## MS treatments

Lopez-Matencio et al. proposed a personalised dosing schedule to reduce the risk of developing PML in patients with elevated Natalizumab serum levels.

Boldrini et al. identified a subset of circulating cytotoxic CD19 B cells expressing serine-protease granzyme B in RRMS patients treated with Fingolimod and Natalizumab, emphasising an antibody-independent mechanism in MS pathology.

Additionally, Walo-Delgado et al. highlighted how baseline blood lymphocyte profiles in MS patients treated with Alemtuzumab could predict AIAEs and help treatment management.

Simona Rolla et al. demonstrated that Alemtuzumab induces a tolerogenic immune response, increasing functional regulatory T cells and reducing inflammatory Th1/Th17 subsets, supporting immune modulation as a therapeutic approach.

Dobreanu et al. found that Cladribine responders showed a depletion of inflammatory Th1, Th1-Th17, and Th17 subsets, which could help to predict positive outcomes.

Research by Pablo Aliaga-Gaspar et al. revealed that increased serum levels of sIFNAR2 in non-responders could be a biomarker for negative IFNβ therapy outcomes.

Fernandes-Velasco et al. demonstrated that Ocrelizumab reduces B and CD20+ T cells and reshapes the T cell profile, especially in non-inflammatory PPMS, suggesting its effects vary with the inflammatory status of patients.

Finally, Mariottini et al. showed that intermediate-intensity autologous hematopoietic stem cell transplantation (AHSCT) significantly reduces serum sNFL levels, indicating decreased axonal damage, particularly in RRMS patients, and underscoring its lasting effect on inflammation-related damage.

## New insight into MS immunopathogenesis

Lomakin et al. demonstrated a delayed maturation of transitional B cells (tBreg) in MS patients, which may contribute to MS pathogenesis. The authors suggested that restoring their maturation and regulatory function could represent a new potential therapeutic strategy.

Several studies have used experimental autoimmune encephalomyelitis (EAE) animal models to explore potential MS treatments. Tang et al. found that T-cell immunoglobulin mucin domain 3 (Tim-3), expressed on immune cells like T cells and macrophages, may inhibit MHC-II-mediated autoantigen presentation and CD4+ T cell activation, influencing EAE outcomes. Targeting the Tim-3/MHC-II signalling pathway could offer new therapeutic options for MS. Hou and Yuki characterised two CCR6+CD4 T cell subsets, showing that one has cytotoxic markers and Th17-related molecules, while another expresses IFNγ and GM-CSF, both contributing to CNS inflammation. These cells could be key targets for future MS therapies.

Hu Lu, Peng-Fei and colleagues indicated that lower levels of fibroblast growth factor (FGF) 23 in the blood are associated with an increased risk of MS, although further investigation is needed to confirm FGF23 as a potential target for treating MS and to understand the mechanism by which FGF23 contributes to the risk of MS.

## Viral cascade and MS pathogenesis

The overview by Meier et al. suggests a central role for Epstein-Barr virus (EBV) in the dysregulation of immune responses and neuronal damage seen in multiple sclerosis (MS). After EBV reactivation in latent B-cells, it could trigger immune complexes and anti-EBNA1-IgG production, leading to a cascade of immune responses involving T, B, NK cells, and cytokines (Type-I IFN, TNF-α, IL-6, -10, -17A). This causes localised inflammation, which, combined with a compromised blood-brain barrier, leads to CNS glia activation and inflammatory lesions. Additionally, this process may activate endogenous viral sequences (MSRV, HERV-K/-W, HHV6A), further intensifying CNS inflammation.

Several studies have highlighted the role of long non-coding RNAs (lncRNAs) in neurodegenerative diseases. Tamizkar et al. found that the differential expression of five specific lncRNAs in the blood can distinguish Parkinson’s disease (PD) patients from healthy donors. In multiple sclerosis (MS), Jalalei et al. identified 52 upregulated, 37 downregulated, and 11 stable lncRNAs in MS patients, suggesting that lncRNA dysregulation may contribute to neuronal death through unknown RNA-based mechanisms. Additionally, bioinformatic analysis by Sabaie et al. revealed that lncRNA SNHG1 might regulate protein processing in T-cell signalling in relapsing-remitting MS (RRMS) patients, although further research is needed to understand the molecular mechanisms underlying neurodegeneration.

## MS biomarkers

Yalachkov et al. highlight the importance of examining peripheral and central inflammation markers at MS diagnosis to understand their connection to depressive symptoms Additionally, investigation from Meng Li et al. suggests that IL-17A, Del-1, and Resolvin D1 may co-regulate MS progression, providing insights into therapeutic targets and patient prognosis. Furthermore, a prognostic model using microarray gene expression data shows promise for effectively classifying MS patients and could serve as a valuable diagnostic tool (Haoran Li et al.).

